# Pediatric emergency backpacks—effects of using xABCDE- and Broselow-systems on pediatric emergency care

**DOI:** 10.1007/s00431-025-06425-w

**Published:** 2025-08-29

**Authors:** Bastian Brune, Doreen Lemm, Maximilian Wolf, Anna Johland, Lars Becker, Cynthia Szalai, Nora Bruns, Thorsten Brenner, Marcel Dudda, Frank Herbstreit

**Affiliations:** 1 Department of Trauma, Hand and Reconstructive Surgery, University Hospital Essen, University Duisburg-Essen, Essen, Germany; 2 Directorate of Emergency Medical Services, Fire Department Essen, Essen, Germany; 3 Fire Department Essen, Essen, Germany; 4 Department of Anesthesiology and Intensive Care Medicine, University Hospital Essen, University Duisburg- Essen, Essen, Germany; 5 Department of Pediatrics I, Pediatric Intensive Care Medicine, Pediatric Neurology, and Pediatric Infectious Diseases, University Hospital Essen, University of Duisburg-Essen, NeonatologyEssen, Germany; 6 C-TNBS, Centre for Translational Neuro-and Behavioral Sciences, University Hospital Essen, University of Duisburg-Essen, Essen, Germany; 7 Department of Orthopedics and Trauma Surgery, BG Klinikum Duisburg, Duisburg, Germany

**Keywords:** Broselow, xABCDE, Pediatric, Emergency medical service (EMS), Backpacks

## Abstract

**Background:**

Pediatric emergencies are a challenge for emergency medical service (EMS) personnel regarding medical expertise and equipment. To address the special requirements in terms of size and weight, attempts are being made to systematically structure pediatric emergency backpacks.

**Objectives:**

The aim of the study was to compare two pediatric emergency backpacks. One structured according to the xABCDE-system, the other to the Broselow-system based on patients’ measurements.

**Methods:**

In total, 115 participants underwent exercises and emergency scenario simulations with two pediatric emergency backpack systems in a prospective, randomized study. First, they performed a material search of six items to test the intuitiveness of both backpack systems. Later, they used the backpacks in the simulation of a pediatric resuscitation and carried out self-assessment and backpack system evaluation.

**Results:**

The handling of the Broselow-system was easier for the participants. Particularly size-specific materials (laryngeal mask (xABCDE vs. Broselow: 61.2 ± 31.3 vs. 24.5 ± 14.2 s, *p* < 0.001)), but also other materials (Magillpliers (37.2 ± 23.3 vs. 7.9 ± 6.1 s, *p* < 0.001), peripheral venous catheter (12.7 ± 5.7 vs. 6.6 ± 2.7 s, *p* < 0.001), pupil lamp (both 17.9 ± 6.9 vs. 16.0 ± 6.9 s, *p* < 0.05)) were found faster. The participants were faster in the resuscitation simulation and selection of large-scale measures using the Broselow-system (e.g. laryngeal mask (187.6 ± 72.6 vs. 155.1 ± 63.5 s, p = 0.001) and preparation of the medication administration in the appropriate dosage (243.0 ± 73.9 vs. 219.8 ± 60.2 s, *p* < 0.05)) and rated the Broselow-system as well as their own performance better in all evaluation categories (*p* < 0.001).

*Conclusions*: As the Broselow-system coded according to size and weight was ranked better in practical application and evaluation, the size and weight-based sorting should be preferred to pure xABCDE systems. The comparison of the different backpack systems should further be tested in more complex simulations and real operations.

**Table:** 

**What is known?** *•In emergency medicine, patient care follows the structured xABCDE approach to prioritize life-saving interventions. This algorithm is established in both prehospital and in-hospital settings.* **What is new?** *• We present the first comparison of two pediatric emergency backpacks structured by the xABCDE algorithm. Both systems were tested in 2 exercises (material search & resuscitation simulation) in randomized order. Participants completed questionaries on the backpacks and their own performance.*

## Background and importance

### General background

Pediatric emergencies are a challenge for emergency medical service (EMS) personnel. Due to the low number of pediatric emergency interventions, the use of pediatric emergency backpack systems is rarely necessary [1]. In addition, there are anatomical and physiological peculiarities that have to be taken into account when treating pediatric patients in time-critical situations [2, 3]. The weight-adapted dosage of medication is a frequent source of error [2].

This initial situation causes specific challenges on both medical expertise and equipment knowledge. Attempts were therefore made to conceptualize pediatric emergency backpack systems intuitively in systematic structure and in terms of size and weight adaption of treatment.

### Pediatric treatment concepts

The xABCDE-scheme is a commonly used treatment concept in emergency medicine. It is used for the structured initial assessment and treatment of both adult and pediatric patients [3]. Many medical measures must be adapted to the size and weight for pediatric patients [3–5]. One possible method for quickly measuring the child's height and estimating body weight is a pediatric emergency tape, which is based on the Broselow-code [6]. This tool was loaded on both backpack systems that were examined.

### Pediatric emergency backpacks

Pediatric emergency backpacks can be sorted into different systematics. The systems contain materials in accordance with the applicable DIN 13232:2011–05 [7]. The system-inherent use of modular bags is common. One of the systems examined is predominantly structured according to the xABCDE-scheme. The modular bags contain all common sizes of pediatric materials. The system is equipped with materials for basic as well as advanced prehospital pediatric care. Emergencies can be handled independently in this system without an emergency backpack for adults.

The other backpack examined is based on the Broselow-system. The modular bags are color-coded and contain all materials for a certain body size range. Some non-size-specific materials for initial care are stored in a separate module bag and in a special compartment of the backpack. Other contents, such as medication, are not carried in the system. It is therefore not possible to work independently with this system. The study participants were provided with an emergency case for adults to supplement the resuscitation simulation, so that medication could be administered from it.

**Fig. 1:**
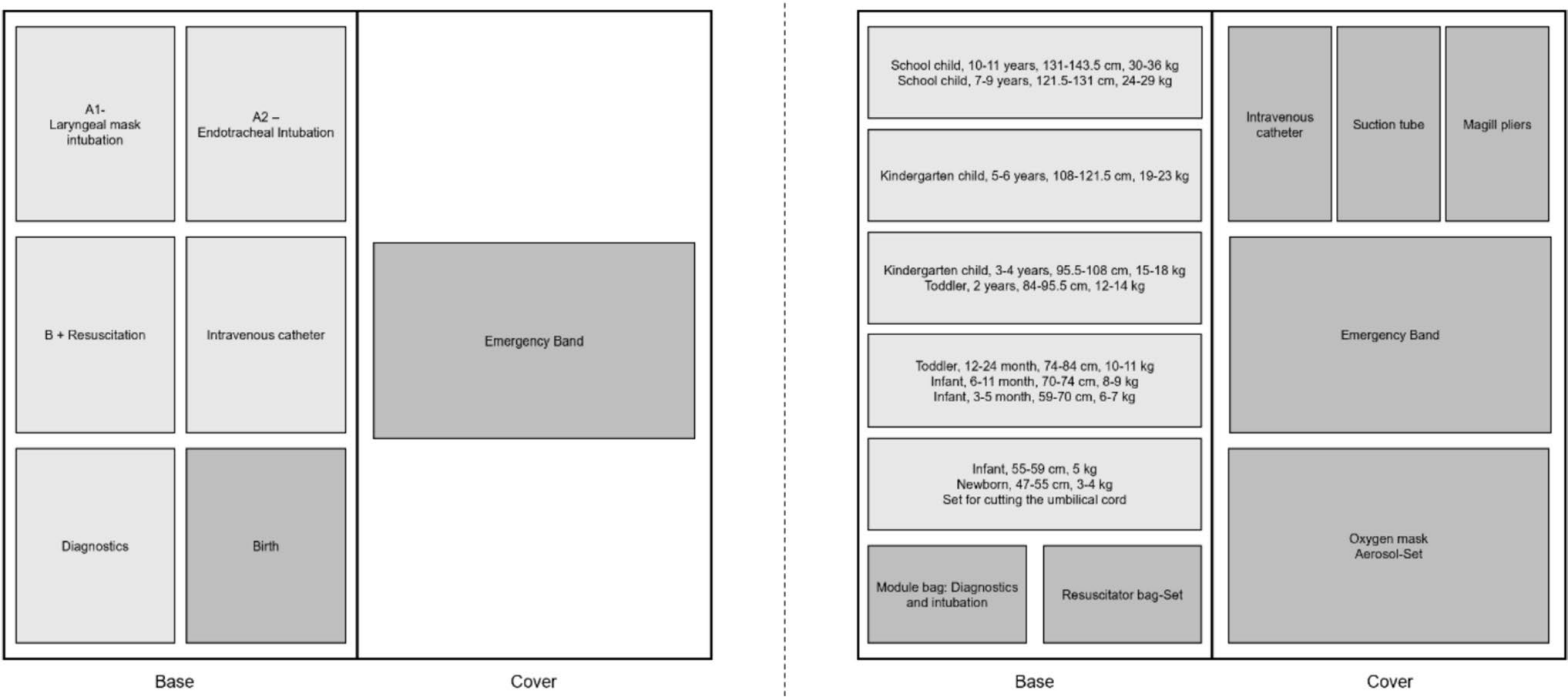
Schematic representation of the backpack systems examined: left, xABCDE-system; right, Broselow system. Module bags that were loaded independently of the scheme are marked in dark

A schematic representation of the pediatric emergency backpack systems can be found in Fig. 1.

## Objectives

In the study, two pediatric emergency backpack systems were compared. The evaluation focused on the intuitiveness of operation and orientation within the backpack systems, which was assessed by measuring the time taken to find and deploy specific materials. In addition, the practical usability of both systems was examined during a simulated resuscitation scenario by recording the time to material application. This was followed by a self-assessment and an evaluation of both systems by the participants.

### Design, settings and participants

The study was conducted in a prospective, randomized controlled study design. The study was approved by the Ethics Committee of the Medical Faculty of the University of Duisburg-Essen (23–11498-BO). It was conducted in accordance with the Declaration of Helsinki.

The participants were recruited from the local EMS, the Essen Medical Faculty and the Essen University Hospital. All participants declared their voluntary participation in writing.

All measurements were carried out in a standardized study procedure. Pseudonymization was implemented to ensure that the questionnaires could be assigned to the practical exercises. All exercises were performed under the assumption that emergency medical measures are time critical.

After a description of the pediatric emergency backpack systems and an introduction to the pediatric emergency tape, the participants were randomized in two groups. Randomization determined which backpack system the participants used first. All parts of the study were completed with both backpack systems.

In exercise 1, the participants had to find items in both backpack systems. Duration was measured independently for each item. The participants were allowed to familiarize themselves with both backpack systems after exercise 1. During exercise 2, a pediatric resuscitation scenario, duration until material search and deployment was measured using a self-programmed stopwatch in Microsoft® Excel® for Microsoft 365 MSO (Version 2407 Build 16.0.17830.20056). All actions performed in the resuscitation scenario could vary depending on the order of execution. As each measure was stopped independently, the subsequent measurement was not affected if a participant did not perform measures or performed them in a different order.

### Excercise 1: Intuitive orientation (material search)

Six frequently used items were selected from most parts of the xABCDE-scheme for both backpack systems. Since the participants did not know the systems in advance, the intuitive application of the systems could be tested. For this purpose, identical materials were selected for both backpacks, including size-dependent and non-size-dependent materials.

### Exercise 2: Simulation scenarios (material application)

A resuscitation of a six-year-old boy was simulated in randomized order of both backpack systems. Since the focus was on the search for equipment and the results should not be distorted by the abilities of the subject, an execution of the measures was not required. Nevertheless, the measures should be performed as realistically as possible so that the duration could be approximated. Duration was stopped for the search and deployment for various items used in respective measures (Table 1).

**Table 1:** Duration was stopped for the following measures during resuscitation

Time until…:
Checking the airway
Five initial ventilations
Start of cardiopulmonary resuscitation
First mask ventilation
First oxygen administration
Intubation prepared
i.v. access prepared
Pediatric tape used
First administration of medication prepared

### Self-assessment and evaluation of the backpack systems

All participants evaluated their performance and the handling of the backpacks after the finalization of the tasks. The evaluation was carried out using a digitized questionnaire on an ordinal scale.

### Outcome measures and analysis

Primary objective of the study was the duration from opening the backpack to finding the required material (exercise 1) or until deployment (exercise 2). Secondary target variables were the evaluation of their own performance and of the backpack systems.

Statistical significance was determined using unpaired two-sided t-tests, Mann–Whitney-U-tests or Pearson´s-chi-squared-tests. A p-value ≤ 0.05 was set as the significance level. Data are given as mean value ± standard deviation.

The data from exercises 1 and 2 were approximately normally distributed. Statistical analyses were made with IBM® SPSS® Statistics Version 29.0.0.0.

## Main results

### Demographics

A total of 115 participants with a medical background volunteered for the study. Most of the test subjects worked as advanced emergency paramedics and basic paramedics, predominantly in pre-hospital care (Table 2). The participants were on average ca. 30 years old (29.5 ± 8.1 years, age range: 18–57 years). 57.4% used a pediatric emergency backpack system in their everyday work, which in 74.2% of cases was not structured according to either the xABCDE-scheme or the Broselow-system. None of the respondents had used a Broselow-backpack system in their daily work.

**Table 2:** Number of participants, sorted by qualifications

	Job title	Participants
EMS personnel	Advanced Emergency Paramedic	30
	Basic Paramedic	39
	Emergency Paramedic Assistant	14
Hospital personnel/students	Physicians	12
	Medical students	20
Total		115

61.7% of the participants had already performed emergency treatment on children, but in most cases only in few (1–10) emergency treatments (40.9%). The self-reported competency when dealing with pediatric emergencies was low overall. 52.2% of the respondents stated that they felt “unsafe” when dealing with pediatric emergencies (“very safe” (1) – “very unsafe” (4) (2.9 ± 0.7)).

### Exercise 1: Intuitive orientation (material search)

**Fig. 2:**
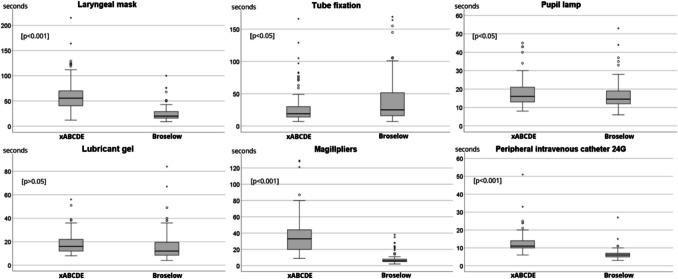
Duration of the material search in seconds

The majority of items were found significantly faster by the participants using the Broselow-system (Fig. 2). When searching for the laryngeal mask for a 1.20 m tall child, there were significant differences in the use of the two backpack systems in favour of the Broselow-system (xABCDE vs. Broselow: 61.2 ± 31.3 vs. 24.5 ± 14.2 s, *p* < 0.001). The pediatric tape was used more frequently when searching for the laryngeal mask in the xABCDE-system (xABCDE vs. Broselow: 79.3% vs. 25.7%, *p* < 0.001). Regardless of the use of the pediatric tape, the subjects were significantly faster in searching for the laryngeal mask in the Broselow-system (pediatric tape used: xABCDE vs. Broselow: 63.8 ± 25.7 vs. 36.8 ± 20.0 s, *p* < 0.001, pediatric tape not used: xABCDE vs. Broselow: 51.8 ± 46.1 vs. 20.2 ± 8.2 s, *p* < 0.05). Significant results were also found in the search for the pupil examination lamp (xABCDE vs. Broselow: 17.9 ± 6.9 vs. 16.0 ± 6.9 s, *p* < 0.05), the Magillpliers (xABCDE vs. Broselow: 37.2 ± 23.3 vs. 7.9 ± 6.1 s, *p* < 0.001) and the search for the 24G peripheral venous catheter (xABCDE vs. Broselow: 12.7 ± 5.7 vs. 6.6 ± 2.7 s, *p* < 0.001).

No significant difference was found in the search for the lubricant gel (xABCDE vs. Broselow: 18.1 ± 8.6 vs. 15.9 ± 12.2 s, p = 0.135). Only the tube fixation was found significantly faster by the participants in the xABCDE-system (xABCDE vs. Broselow: 28.6 ± 25.7 vs. 39.2 ± 34.9 s, *p* < 0.05).

### Exercise 2: Practical application of the backpack systems in a pediatric resuscitation simulation

The initial resuscitative actions were performed by the participants with both backpack systems at approximately the same time (airway control (xABCDE vs. Broselow: 11.9 ± 5.0 vs. 12.1 ± 6.3 s), 5 initial breaths (xABCDE vs. Broselow: 55.3 ± 30.3 vs. 59.4 ± 29.1 s), chest compressions (xABCDE vs. Broselow: 43.7 ± 30.8 vs. 44.8 ± 31.6 s), start of mask ventilation (xABCDE vs. Broselow: 79.0 ± 31.0 vs. 81.3 ± 28.4 s), oxygen administration (xABCDE vs. Broselow: 102.8 ± 49.3 vs. 101.8 ± 44.7 s), preparing a peripheral venous catheter (xABCDE vs. Broselow: 199.6 ± 68.1 vs. 190.6 ± 50.3 s) (all p > 005)).

**Fig. 3:**
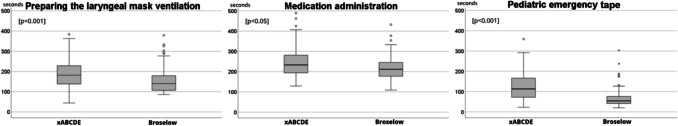
Duration for measures recorded during the resuscitation simulation in seconds

In the later stages of resuscitation, the required materials were found more quickly in the Broselow backpack system. The Broselow-system resulted in significantly greater time savings in the course of size-dependent measures, especially in preparing the appropriate size for laryngeal mask ventilation (xABCDE vs. Broselow: 187.6 ± 72.6 vs. 155.1 ± 63.5 s, p = 0.001) and medication in the appropriate dosage (xABCDE vs. Broselow: 243.0 ± 73.9 vs. 219.8 ± 60.2 s, *p* < 0.05). During resuscitation, the pediatric band was used significantly faster in the Broselow-system (xABCDE vs. Broselow: 124.9 ± 69.8 vs. 67.0 ± 44.1 s, *p* < 0.001) (Fig. 3).

### Evaluation of the backpack systems

**Fig. 4:**
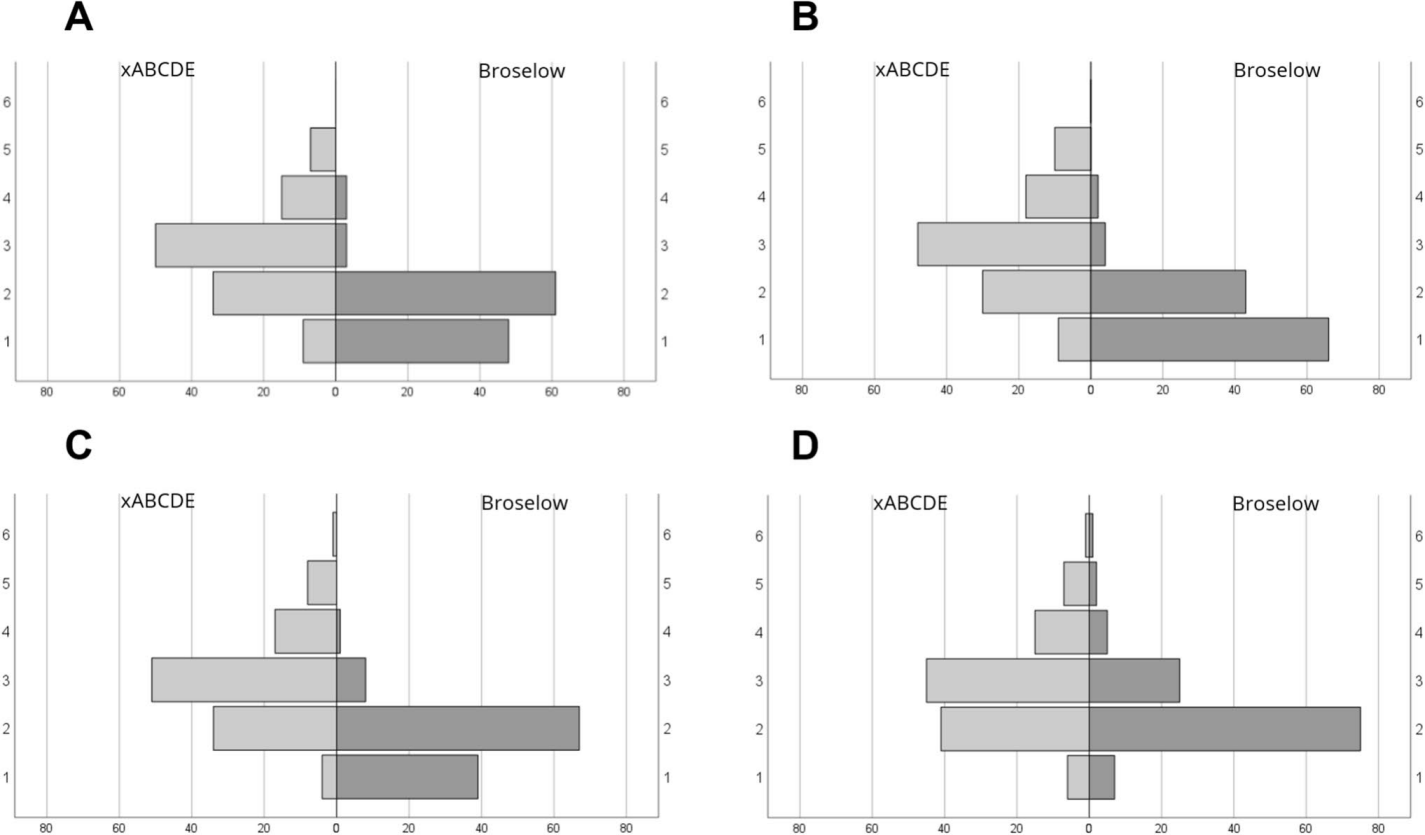
Evaluation of the participants ‘ opinions. (*x*-axis: number of participants, **A** module bag composition, **B** consistency, **C** overall backpack system, **D** own performance). (1) very good/satisfied, (2) good/satisfied, (3) less good/satisfied, (4) rather poor/unsatisfied, (5) poor/unsatisfied, (6) very poor/unsatisfied

The evaluation of the backpack systems showed that the participants rated the Broselow-system significantly better (Fig. 4). They were significantly more satisfied with both the composition (*p* < 0.001) and the consistency of the backpack system (*p* < 0.001). The overall rating of the backpack systems was also significantly better for the Broselow-backpack system (*p* < 0.001).

**Fig. 5:**
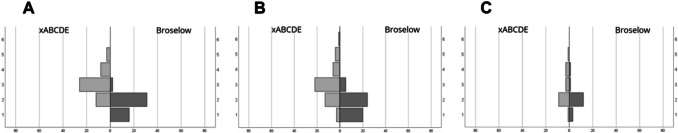
Evaluation of the participants ‘ opinions depending on their material working routine. (*x*-axis: number of participants, **A** no case, **B** neither xABCDE nor Broselow, **C** xABCDE). (1) very satisfied, (2) satisfied, (3) less satisfied, (4) rather unsatisfied, (5) unsatisfied, (6) very unsatisfied

17 participants used a backpack system based on the xABCDE-scheme in their everyday work to treat children. The use of an xABCDE-coded system in everyday work did not lead to the participants rating this backpack system better. The remaining 98 participants, who did not use a pediatric emergency system in their everyday work or used a system that was structured according to neither the xABCDE-scheme nor the Broselow-system, rated the Broselow-coded backpack system better (*p* < 0.001). This result is independent of whether the participants already used a pediatric emergency system that was structured according to neither the xABCDE-scheme nor the Broselow-system (49 participants) (*p* < 0.001) or did not yet use a pediatric emergency system in their everyday work (49 participants) (*p* < 0.001) (Fig. 5).

The use of the backpack systems influenced the perception of the participants' own performance. When working with the Broselow backpack system, the participants rated their own performance significantly better (*p* < 0.001).

## Discussion

### Exercise 1: Intuitive orientation (material search)

Pediatric emergencies are often treated by non-specialized pediatric first responders who have limited experience in dealing with pediatric emergencies and are not very application-experienced with pediatric equipment [8, 9]. Adapting equipment, measures and medication dosage to the child's height and weight plays a particularly important role in the care of pediatric patients and is a source of error [2, 3]. Intuitive operation is therefore even more important in pediatric patients than in other areas of prehospital emergency care due to the rarer use of equipment and the existing stress, all with the aim of reducing errors.

In the study the first task in the material search was to deploy the laryngeal mask, a particularly suitable item that is used in time-critical situations in the preclinical setting and must be found quickly in the correct size. This is because the oxygen requirement of infants and young children is approximately twice that of adults [10]. Therefore, the rapid establishment of a secure airway for oxygenation is particularly important. The transition from a compensated to a decompensated respiratory state in pediatric patients is often unpredictably rapid [3, 9], and the time to fall below critical thresholds is often significantly reduced compared to adults [11]. The collected data indicate that participants were able to identify and apply the laryngeal mask significantly faster when using the Broselow-system. Considering the risk of rapid respiratory decompensation, this time advantage may hold substantial clinical relevance.

The application of a laryngeal mask can be understood as a continuation of bag-mask ventilation [12, 13]. Endotracheal intubation is only recommended for emergency physicians (EPs) providing assistance or to personnel that has sufficient experience. In Germany most users should prefer to use the laryngeal mask to achieve improved oxygenation [5]. In this study, the Broselow-system demonstrated a significant and clinically relevant advantage in the search for and application of size-specific laryngeal masks. The significant differences from exercise 1 remain in exercise 2 (xABCDE vs. Broselow: 63.8 ± 25.7 vs. 36.8 ± 20.0 s, *p* < 0.001 and 187.6 ± 72.6 vs. 155.1 ± 63.5 s, *p* < 0.001) and will be discussed afterwards.

In the xABCDE-system, manual determination of all material sizes is required. This explains the significantly more frequent use of the pediatric tape for size-adaptive selection of the laryngeal mask in the study. In contrast, in the Broselow-system, the module pockets are labelled according to the size and weight of the child, which facilitates targeted material removal even without prior use of the tape.

The other items to be searched for are frequently used materials for pediatric emergency care. Items from almost all parts of the xABCDE-scheme were queried [3, 9]. In particular, size-specific items, but also some non-size-dependent materials, were found significantly faster in the Broselow-system. Items for which no time advantage was found in the Broselow-system were not sorted by size according to the Broselow-code and were located in the supplementary module bag (tube fixation, lubricant gel). When evaluating these items, the problem of multiple storage, which would be necessary in the module bags according to Broselow, is a limiting factor.

### Exercise 2: Application in a simulation scenario

Pediatric resuscitation differs from adult resuscitation in many ways. Unlike in adults, circulatory arrest is not usually caused by a cardiac problem but is mostly the result of a respiratory disorder that causes hypoxia [10].

In order to address the primary cause directly and replenish the oxygen reserves, ventilation is given greater priority than in adult resuscitation. Five initial breaths are administered at the beginning of the resuscitation. They are followed by a resuscitation rhythm of 15 chest compressions to 2 breaths [3, 9]. Due to the significantly shorter time between ventilations, the medical team, has significantly less time to find the necessary equipment and adjust it to the child's size and weight, especially in a two-person resuscitation. The shorter time available for searching and implementing measures could lead to additional stress, uncertainty, and decreased performance among inexperienced and uncertain personnel in the xABCDE system. These effects may be less pronounced in the Broselow system.

The measures carried out in the initial phase of the resuscitation simulation did not necessarily require the use of materials from the backpack systems, which is why the lack of difference was to be expected. As the simulation progressed, the participants found the materials in the Broselow-system significantly faster, especially when performing size-dependent tasks. In the short intervals of the pediatric resuscitation algorithm in a 15:2 rhythm, the more intuitive system may offer a better starting point for finding materials. This aspect is particularly important in pediatric resuscitation to reduce hands-off time to a minimum and thus improve the outcome [3, 9].

The results of the study showed the benefits of early use of the children's emergency tape in the practical application of the Broselow-system. As all size-dependent materials are pre-sorted accordingly, the tape made efficient selection of size-adapted equipment possible by directly measuring the child's size.

The use of pediatric emergency tape is discussed controversial. Numerous studies support the effectiveness of this tool in estimating a child's weight and size, thereby facilitating appropriately sized interventions and accurate medication dosing [14, 15]. Pediatric tapes offer more accurate weight estimations than age-based formulas or visual assessments by experienced rescuers, which can help reduce medication errors [9]. But critics highlight significant limitations. For instance, the discussion addresses whether dosing according to actual body weight is preferable to dosing according to ideal body weight. Concerns have been raised regarding the potential inaccuracy of these length-based estimates of actual weight, particularly for children whose weight deviates substantially from age- or length-based norms, which may lead to dosing errors [16, 17]. Nevertheless, length-based weight estimates, such as the pediatric emergency tape, should be used for quick estimates of a child's weight [3].

### Questionnaires and self-assessment

#### Evaluation of backpack systems

Structured handling of materials is important to enable timely patient care. Successful management of complex emergency situations can only be achieved if logically structured, easy-to-use material organization is available [18]. The participants rated the material organization of the Broselow-system significantly better in all categories. This evaluation confirms the data collected and the conclusions drawn from it. The participants experienced clear advantages when working with the Broselow-system. The Broselow-system was also rated better by participants who had already worked with an xABCDE coded system.

#### Self-assessment

In addition to the lack of routine due to the infrequency of calls, special considerations must be considered treating children. In addition to that, pediatric emergency missions are often emotionally stressful. These circumstances can lead to uncertainty and anxiety, especially in very young patients [8]. The improved performance using the Broselow-system suggests that the structure provides certainty in the treatment of pediatric emergencies. Taking into account the Yerkes-Dodson law, which states that increased anxiety and stress – for example as a result of uncertainty in the emergency care of pediatric patients – can significantly impair performance [19], it can be deduced that greater satisfaction of the participants with their own performance could contribute to a qualitative improvement in emergency care. This effect may be more pronounced in real-life operations due to the greater stress compared to simulation training [20]. In addition to the measurable quantitative effects, a qualitative improvement in pediatric emergency care when working with the system is therefore also conceivable.

### Limitations

The study was carried out a data collection in the context of an exercise and a simulation. This means that statements on real-life use can only be assumed to a limited extent and cannot be made on the quality of implementation. Qualitative interpretations are not possible, since the measures were not implemented realistically by the inhomogeneous collective.

Due to the fact that the data was only collected in Germany, it is only partially possible to make general statements. In particular, geographical differences in handling pediatric emergencies or other standard operating procedures and treatment algorithms, especially in differing international EMS, may require differently composed backpacks or materials.

In the case of the participants who did not perform pediatric resuscitation in accordance with the guidelines, the measures were carried out in different orders, which also led to a distortion of the results.

The transfer of the results to real-life situations is only possible after an individual analysis of the pediatric emergency backpacks with other materials in the emergency services or hospital.

## Conclusion

Although the xABCDE-system is predominantly used for the care of patients in everyday practice, the Broselow-system, which is coded according to height and weight, enabled most study participants to provide faster and more structured material handling in the emergency care of children. The study participants rated both the Broselow-system itself and their own performance better with this system. When selecting pediatric emergency backpack systems, size and weight-based sorting could be advantageous compared to pure xABCDE sorting to make intuitive, rapid treatment possible.

The comparison of different backpack systems should also be carried out in more complex simulations and real operations. Given the diverse professional backgrounds of the participants, a differentiated evaluation according to occupational groups could be useful to better identify possible group-specific differences.

## Data Availability

No datasets were generated or analysed during the current study.

## Abbreviation

Broselow: Backpack system, which is mainly based on the Broselow code
DIN: DIN standard, Standard specification from the German Institute for Standardization
e.g.: Exempli gratia / for example
EMS: Emergency medical service
EP: Emergency physicians
G: Gauge
s: Seconds
vs: Versus
xABCDE: Backpack system, which is mainly based on the xABCDE scheme
